# Environmental Monitoring: A Comprehensive Review on Optical Waveguide and Fiber-Based Sensors

**DOI:** 10.3390/bios12111038

**Published:** 2022-11-17

**Authors:** Muhammad A. Butt, Grigory S. Voronkov, Elizaveta P. Grakhova, Ruslan V. Kutluyarov, Nikolay L. Kazanskiy, Svetlana N. Khonina

**Affiliations:** 1 Samara National Research University, 443086 Samara, Russia; 2 Ufa University of Science and Technology, Z. Validi St. 32, 450076 Ufa, Russia; 3 IPSI RAS-Branch of the FSRC “Crystallography and Photonics” RAS, 443001 Samara, Russia

**Keywords:** environmental monitoring, optical waveguide, optical fiber, photonic sensors, gas sensing, water quality monitoring, volatile organic compounds

## Abstract

Globally, there is active development of photonic sensors incorporating multidisciplinary research. The ultimate objective is to develop small, low-cost, sensitive, selective, quick, durable, remote-controllable sensors that are resistant to electromagnetic interference. Different photonic sensor designs and advances in photonic frameworks have shown the possibility to realize these capabilities. In this review paper, the latest developments in the field of optical waveguide and fiber-based sensors which can serve for environmental monitoring are discussed. Several important topics such as toxic gas, water quality, indoor environment, and natural disaster monitoring are reviewed.

## 1. Introduction

Environmental monitoring includes all procedures and actions taken to assess the state of the environment. The previous century has seen significant advancements in the field of environmental monitoring, but the on-site control of toxins remains a challenging issue. In particular, the requirement for effective early warning systems is expanding along with the number of polluting sources [1]. The management of environmental deterioration and the preservation of the natural environment’s quality for the benefit of future generations depend on real-time and on-site pollution monitoring. The traditional analytical methods, which are based on chromatographic [2] and spectroscopic technologies [3], continue to be the most effective for controlling the environment because of their precision and sensitivity. Nevertheless, these procedures are restricted to centralized laboratories, call for pricey equipment, take a lot of time, and necessitate experienced workers. Optical sensors and photonic devices have recently come to light as intriguing alternatives to conventional analyses’ exorbitant costs and slow speed. Due to their speed, specificity, sensitivity, reusability, ability to permit permanent and unattended operation in the field, and ability to provide the portable analytical instruments and early warning systems that are required [4].

Environmental risks pose a threat to both humans and the things we consider important. Even though they might have a variety of diverse sources, we typically conceive of dangers as arising from the interaction of human, technical, and natural systems. As a result, we frequently categorize hazards based on the events that cause them: rare natural occurrences (such as earthquakes and hurricanes) [5] and relatively common ones (such as blizzards and coastal erosion), extreme technological occurrences (such as nuclear power plant accidents or chemical spills), unrest and armed conflict, biological agents, or biohazards (epidemics, infestations, and bioterrorism), and chronic and globally significant hazards. Floods are the most frequent environmental hazard, damaging more than earthquakes, volcanic eruptions, and tsunamis combined [6]. Flooding may be an attribute of a complex of dangers during extreme weather events that also include mudslides, gales, and tidal surges and causes much more havoc. Floods are the worst form of danger since they often occur, have a wide-ranging impact on every region of the Earth, and are monster threats that do not simply happen once in a lifetime [7]. Low-magnitude, frequent floods can harm farmland, disrupt, or destroy buildings and infrastructure, stop business and other operations, expose people to health concerns, and so forth.

An appropriate supply of high-quality water is essential for health and is a fundamental human right. However, drinkable water is becoming extremely scarce [8]. In special, the health dangers caused by dirty water sources are more widespread and more harmful in poorer nations than in wealthy countries [9]. Surface water may be contaminated by sewage, industrial water discharge, or runoff from land clearing [10]. Ground water can also be poisoned by saltwater intrusion or waste dumping sites [11]. Water supplies were not always continuously assessed and investigated [12]. It takes sophisticated monitoring techniques to obtain evidence about pollutants in the environmental ground and surface water, safeguard water quality, and enhance the quality of household water supplies [13].

Due to recent developments in the creation of portable, less expensive air pollution sensors reporting data in near real time at a high time resolution, improved computational and visualization capabilities, and wireless communication/infrastructure, the framework for air pollution monitoring is quickly changing. By strengthening compliance monitoring and complementing ambient air monitoring, these developments could assist conventional air quality monitoring. With the use of sensors, communities and individuals are starting to have the knowledge they need to understand their environmental exposures. With these data, community- and individual-based pollution reduction plans can be devised, and connections to health markers can be understood.

**Figure 1:**
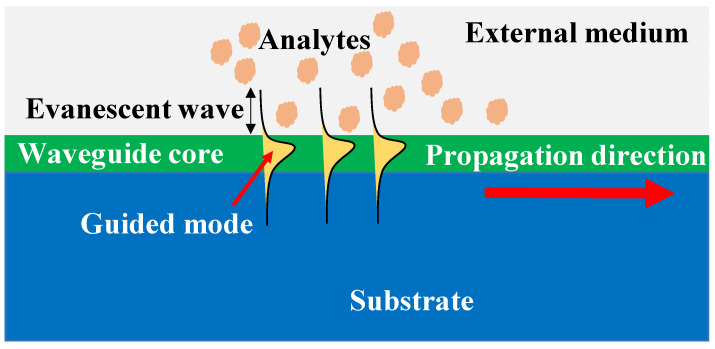
Illustration of evanescence field sensing.

The evanescent wave sensors track variations in the refractive index [14,15,16,17]. To produce a propagating or localized electromagnetic mode, these sensors make use of the electromagnetic waves’ confinement in a dielectric and/or metal structure [18]. A portion of the restricted light spreads to the surrounding medium, producing the evanescent wave. Through this evanescent wave, refractive index shifts in the external medium cause a local change in the excited electromagnetic mode’s optical characteristics, most notably a variation in the effective refractive index [19]. When a receptor layer has been mounted on the surface of the guiding structure, the exposure of the functionalized surface to the complementary analyte and the ensuing (bio)chemical interaction between them causes a local change in the refractive index. The interaction may be quantified by correlating its amplitude with the concentration of the analyte and the interaction’s affinity constant. Only changes near the sensor surface will be detected since the evanescent wave only approaches the exterior medium up to hundreds of nanometers and decays exponentially; as a result, the background from the external medium will be minimally affected. The ability to work under a label-free scheme gives evanescent wave sensors several benefits over other types of sensors, such as fluorescence sensors [20]. This results in a quicker and more affordable total detecting process. The scheme of the evanescent wave label-free sensing is shown in Figure 1. Labels may also have an impact on how the receptor and analyte interact, which may result in decreased performance when analyzing a complicated matrix. Other benefits of photonic sensors are their resilience to electromagnetic interferences, high sensitivity, wide bandwidth, and, most significantly, their potential to be made smaller and more portable thanks to the scalable technologies used in their production.

The goal and advantages of environmental monitoring are to determine if the environmental quality is improving or declining. Making choices for governmental and non-governmental organizations requires a lot of information, which is obtained by environmental consultants by monitoring the environment. Observing and analyzing trends and patterns in the presence of air pollutants in the atmosphere is the primary goal or advantage of environmental monitoring. The purpose of environmental monitoring varies depending on the circumstance, but it is crucial to ensure that businesses comply with environmental laws and regulations, assess the effectiveness of recently installed machinery, and monitor employees’ health. It assists in identifying threats to people and wildlife, determining the potential for population movement from high-density to low-density regions, and limiting gas emissions. The following are a few of the benefits of environmental monitoring:

√- The people who treat patients and raise public knowledge of the condition and its management strategies are healthcare professionals. They are also worried about how a certain initiative may affect the environment, for example. high levels of noise, poor air quality, etc. They are also worried about the long-term and short-term impacts of pollution on the ecosystem and human health.
√- When engineers design the new seaside motorway, they should be aware of potential sea level rise, the degree of vibration at the ocean’s bottom, and any other environmental elements that could have an impact on the strength of the bridge, so that they may take safety precautions when building a bridge.
√- It is crucial to disseminate precise information about the location, timing, and severity level when a tsunami or earthquake strikes in a certain region so that aid may be sent promptly to the designated time and location. The advantages of environmental monitoring also include the ability to anticipate and respond to such incidents.
√- Environmental monitoring data such as severe rainfall, cyclones, and tsunami may be used by farmers, foresters, hunters, and fishermen to plan their activities. The intensity of the natural danger can be reduced if they are warned. Additionally, farmers may learn about their soil’s fertility so they can utilize the necessary fertilizers to increase production.
√- Big industries must be aware of the kinds of pollutants and quantities released from their facilities. They require environmental monitoring data and take environmental pollution reduction measures; hence, they should be included in benchmarks.
√- Data from population monitoring are used by the government to make community mobilization decisions. The government may opt to relocate some industrial districts to low-density regions if a specific city has a high population density and is experiencing issues with the availability of water, energy, and, most crucially, space and land. Additionally, the government builds new communities by utilizing the information gathered through environmental monitoring.

**Figure 2:**
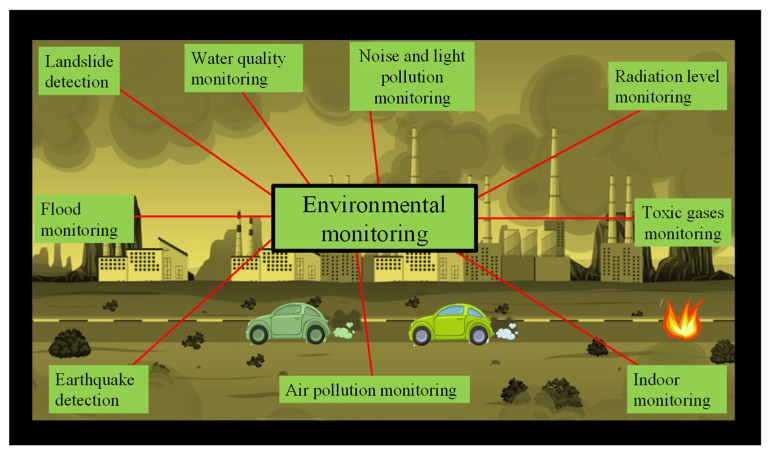
The environmental monitoring applications.

Scientific research and environmental monitoring are closely intertwined. For instance, the government can intervene if the environmental monitoring program identifies a specific contaminant that has the potential to harm aquatic or terrestrial species. Additionally, it can spark an investigation into how that contaminant affects people, animals, or aquatic life so that remediation techniques might be developed. Environmental monitoring includes keeping an eye on the human population, as well as the air, water, soil, and land. Additionally, it aids in recognizing environmental stress, comprehending environmental trends, and assessing the success of initiatives and programs. The environmental monitoring applications are shown in Figure 2.

To manage resources such as oil, natural gas, arable land, and others economically, safely, and successfully, environmental monitoring is crucial. Various green technologies have been created to achieve this. Understanding and anticipating environmental changes, nevertheless, remain difficult tasks. As a result, it is important to keep an eye out for sudden changes that threaten the environment or public health [21]. Analyzing the chemical and physical characteristics of surroundings has been the focus of monitoring technology. As per analytes [22,23] or sensing principles [24,25,26], electrochemical sensors have been created as monitoring sensors in a variety of research. They benefit from low power consumption, good selectivity, and a linear connection between output signals and analyte content, among other things [27]. In most electrochemical sensors, the electrodes are covered in a catalyst that encourages the oxidation or conversion of particular chemical species [28,29]. The research of catalyst species with high durability in severe settings, even though they include such high-selectivity catalysts, proves difficult. As monitoring sensors in challenging situations, thermoelectric and electromechanical sensors, for example, have also been investigated.

The main environmental reason for death is air pollution [30]. The primary pollutants (nitrogen oxides (NO_x_), ozone (O_3_), volatile organic compounds (VOCs), and tiny particulate matter (SP)) emission levels are now steady in most major European cities, which is a troubling development. Reducing and tracking air pollution goes beyond only the effects on the environment and affects public health. Considering this sobering finding, it is essential to guarantee that the presence of these contaminants in ambient air is continuously measured to apply restoration measures and procedures for treating, destroying, or trapping discharge gases.

A microelectromechanical sensor has received a lot of attention due to its tiny size and affordable fabrication [31]. Due to failures brought on by corrosion, electrical disturbance, creep and plastic deformation, and hostile conditions, such sensors have a limited lifespan. It is essential to build sensors that are robust in tough settings for lengthy operating times, particularly since environmental monitoring sensors must function for extended periods in challenging situations. Systems of heuristic quality control will concentrate on effective behavior and environmental constraints on a continuous, real-time basis to identify potential failures at an early stage and create better a sensor platform that promotes better reliability tests and environmental monitoring methods. Due to their tiny size, low power consumption, and perhaps cheap manufacturing costs, wireless sensors—also known as “nodes” or “motes”—are appealing for this application [32]. Additionally, they are simple to question, and connection between units is employed to create a network that collects all the data necessary in real-time and either transmits it or, if attached to a memory, retains it for later use. Sensors can be interfaced with a variety of wired or wireless communications interfaces as necessary, based on the desired application [33,34].

Due to their compact size, versatility, and affordable production, microelectromechanical systems (MEMS) have the potential to significantly contribute to the shrinking of wireless sensor nodes. It may also be possible to create sensor redundancy because of the manufacture of numerous MEMS sensors on a single substrate, allowing for better degrees of integration and dependability. MEMS sensors offer several benefits, particularly in the context of Health and Usage Monitoring Microsystems [35]. The ability to correct cross-sensitivities and the potential for deploying the resulting chip in a larger modular system where sensed data can either be logged or immediately processed make MEMS-based sensor technology a rapidly expanding field with a promising future in a broad range of uses.

The usage of optical fiber sensing techniques has expanded over the past 30 years to a wide range of applications in several scientific fields. With the introduction of realistic, low-loss single-mode fiber created for the telecommunications sector in the middle to late 1980s, the technology, and its specialized instrumentation began to appear as a separate subject. This made it possible to communicate across great distances and to sense environmental variables. For hostile conditions, the optical fiber sensor is a feasible sensor that employs optical cables instead of electric wires because they have less heat loss and a higher data bandwidth than electrical sensors [36]. Extensive optical connections enable interconnection over distances of hundreds of thousands of kilometers. Submarine cables installed all over the world have enabled the connection of vast information technology resources from continent to continent. Because optical fiber has special properties including immunity to electromagnetic interferences and chemical corrosion as well as minimal heat loss, optical fiber cables guarantee durability even under the oceans for extended periods [37]. This shows that the optical fiber sensor can also produce and transmit measurement data with high sensitivity even under challenging environmental circumstances, as those seen in nuclear power plants [38,39] and downhole oil recovery wells [40]. Additionally, the optical fiber sensor is compliant with the latest fiber-optic communication infrastructure, allowing for remote management of environmental surveillance using a network operating system.

Electrochemical and optical sensors have seen rapid advancement in recent years [41,42]. Their usage is becoming simpler, and the range of potential uses is expanding, notably for measuring air quality. The development of sensing devices has altered how many different air contaminants are monitored, enabling quick online readings in dense networks. The cost of air quality measuring technology has decreased, making it more generally accessible. Although the usage of sensors is ubiquitous, it is difficult to determine the accuracy of the measurements, and because of the swift advancement of the technology, it is also difficult to predict long-term performance [43].

## 2. Types of Environmental Monitoring Optical Sensors

In this section, recent advances in optical sensors for four critical environmental parameters are discussed.

### 2.1. Toxic Gas Monitoring Sensors

For environmental and safety considerations, there is now a lot of curiosity about the realization of optical fiber gas sensors [44,45,46]. Compared to typical sensors, optical fiber systems have benefits including intrinsic safety from their non-electrical nature, remote access to dangerous locations, and the possibility of distributed or quasi-distributed systems. Regretfully, the essential characteristics of gases’ vibrational absorption are often found in the infrared (IR) region, outside the silica fibers’ transmission window. However, several gases of relevance also feature overtone and combination absorption lines in the near-IR (λ = 1–2 μm), which may be identified by silica fibers and LED or laser diode sources [47,48]. Examples include methane (CH_4_), carbon monoxide (CO), carbon dioxide (CO_2_), and hydrogen sulfide (H_2_S). Future advancements in mid-IR sources, fibers, and detectors will be crucial as these lines are often 200 times weaker than the basic absorption characteristics.

#### 2.1.1. Optical Fiber-Based Gas Sensors

Photonic crystal fiber (PCF) has made significant progress in optical sensing during the past few decades. The area of fiber optics is no longer restricted to telecommunication applications because of advancements in optical instrumentation. PCF sometimes referred to as holey fiber, is made up of tiny cylindrical air holes that are organized at periodic intervals and run the whole length of the fiber. The typical PCF is built of fused silica, which has a symmetrical pattern of air holes or voids that run perpendicular to its axis. In contrast to conventional optical fibers, this one has a single material that serves as both the core and cladding. The development of opto-devices and sensors has paid significant attention to PCFs because of their distinctive features, including design flexibility, the ability to regulate light, quicker detection speed, and a structure that can be downsized. Additionally, by adjusting the structural characteristics of PCFs, such as the size of the air holes, the pitch, and the number of rings, the evanescent field can be regulated, which opens a wide range of potential applications, particularly in sensing. Since it offers such a wide range of uses, including filters [49], switches [50,51], electro-optical modulators [52], polarization converters [53], and sensors [54,55], PCF has recently got a lot of interest. Since a few decades ago, PCF has been explored extensively as a potential contender for optical sensing. To address safety concerns, extremely sensitive liquid and gas sensors are crucial in industrial operations, particularly for spotting poisonous and combustible gasses or liquids. Enhancing the performance of liquid and gas sensors has therefore emerged as one of the serious issues. Liquid and gas detection based on photonic crystal fibers have demonstrated reliable results in terms of sensitivity responsiveness. Researchers have recently shown a lot of interest in the creation of a PCF-based system for detecting the environment and public safety [56,57].

**Figure 3:**
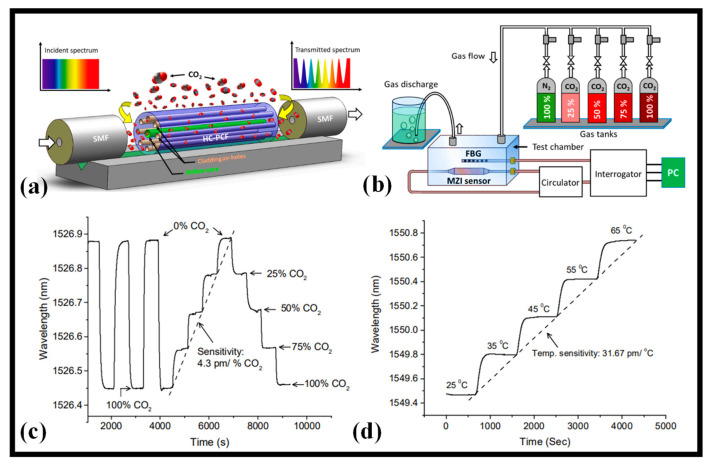
HC-PCF-based gas sensor, (**a**) schematic of MZI developed using a small stub of HC-PCF [58], (**b**) experimental schematic for sensor characterization and interrogation [58], (**c**) the response of the sensor to CO_2_ gas concentration [58], (**d**) the response of the sensor to temperature change [58].

For environmental monitoring applications, a hollow core photonic crystal fiber (HC-PCF)-based MZI is presented that can detect ambient CO_2_ levels as shown in Figure 3a [58]. A stub of HC-PCF was sandwiched between a lead-in and lead-out SMF to create the device. The sensor was packed and described for specified CO_2_ gas concentrations. The sensor exhibits a linear response to CO_2_ levels with a sensitivity of 4.3 pm/% CO_2_ at atmospheric pressure and room temperature. The precision of the sensor is 0.2% CO_2_ given that the measuring tool employed in this investigation has a wavelength stability of 1 pm. The sensor exhibits quick reaction and recovery times of 64 and 69 s, respectively, for the test chamber dimension of 14.5 × 11.2 × 4.4 cm. The sensor was enclosed in gas-permeable, water-resistant membranes. The sensing apparatus showed how to monitor CO_2_ levels in both subterranean and aquatic conditions and detect leaks at laboratory levels. With remarkably quick reaction and recovery times, the sensor demonstrated consistent and accurate detection of CO_2_ concentrations. The schematic presented in Figure 3b is used to characterize CO_2_ gas concentration in near atmospheric pressure [58]. The fiber-optic technology has promise for monitoring CO_2_ content and leakage in the environment, according to the experimental research of this work [58]. A fiber Bragg grating (FBG) was also inserted in the chamber to track any temperature changes that occurred throughout the tests. Figure 3c illustrates how the sensor exhibits a regular pattern of changes in response to varying CO_2_ gas concentrations. Figure 3d depicts the sensor’s reaction to a change in room temperature between 25 °C and 65 °C. With a sensitivity of 31.67 pm/°C, the sensor exhibits a uniform spectral shift with temperature change [58]. A few important works on optical fiber-based gas sensors are presented in Table 1.

#### 2.1.2. Optical Waveguide-Based Gas Sensors

However, the mid-infrared (mid-IR) spectral region has recently gained a lot of attention, since it holds the potential of realizing several intriguing possibilities [71,72] due to the presence of absorption peaks of many trace gases, including CO, CO_2_, NO, NH_3_, and CH_4_, among others [73,74]. These gases experience a significant absorption in the mid-IR region > 2.5 μm because of the basic rotational and vibrational transitions. Recently, several gas sensors based on various platforms and technologies have been presented, including electrochemical sensors [75], the spectroscopic approach [76], and gas chromatography [77], among others. Most of these sensor systems are big and typically pricy. They are thus not practical or user-friendly. The fundamental building block of a wide range of optical sensors is a nanophotonic waveguide [78]. In this situation, optical waveguides built on the SOI platform provide an attractive substitute that uses evanescent field absorption sensing to find trace gases. Evanescent field absorption-based gas sensors are only effective when the gas being measured exhibits the characteristic absorption line at the relevant wavelength. Additionally, there is a connection between the optical attenuation at a certain wavelength and the gas content. Numerous gas sensors based on optical waveguides [14,17,79,80] and optical fiber [81,82] have been developed to operate on this phenomenon.

**Figure 4:**
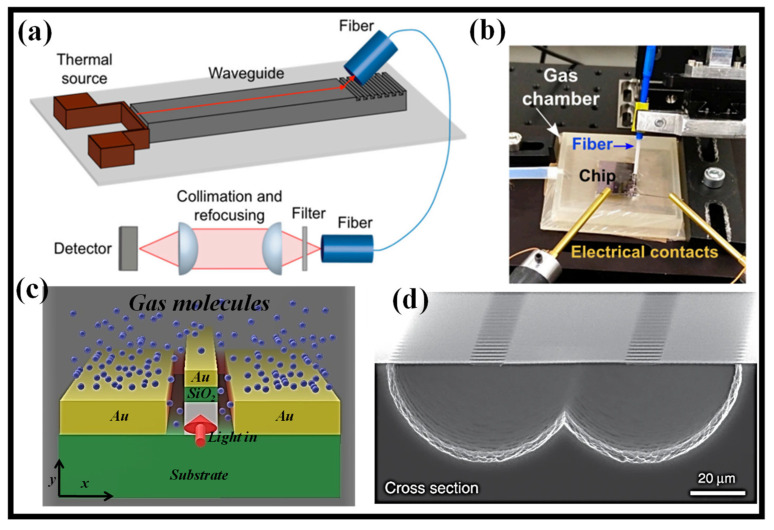
Recently proposed waveguide-based gas sensors, (**a**) schematic view of the gas sensor measuring setup [78], (**b**) experimental realization of the gas cell [78], (**c**) schematic of the polarization independent hybrid plasmonic waveguide for gas sensing [84], (**d**) SEM image of the suspended rib waveguide [86].

Recently, there has been a spike in demand for gas detectors that are miniaturized and CMOS compatible. Optical techniques may be quicker and more reliable than sensors that use metal-oxide chemical interactions. A combination of an external laser source and silicon waveguides built using CMOS technology has lately been used to perform CO_2_ detection by evanescent-wave absorption in the mid-infrared [83]. In [78], it is shown that a low-cost integrated heat source can detect CO_2_ at concentrations as low as 3%. A schematic of the proposed gas sensor and measuring setup is shown in Figure 4a [78]. A compact gas container (Figure 4b) containing the sensor chip is continually flushed with a combination of CO_2_ and N_2_ gas. Two openings on the top of the chamber serve as the departure points for the gas and are designed to receive the optical fiber and electrical connections. A mass-flow control device regulates the concentration of the two gases in the mixture, and the gas moves at a rate of 100 mL/min. By lowering the flow speed to lesser values, no difference in the measured transmittance was seen. New designs are now being developed to raise the still relatively low sensitivity since these results are encouraging for future technical advancements toward on-chip mid-infrared photonic gas sensors.

It is suggested to use a hybrid plasmonic waveguide (HPWG) with a polarization-insensitive design that is tuned to the methane gas absorption line at 3.392 μm wavelength [84]. The schematic of the waveguide is shown in Figure 4c. For both transverse electric (TE) and transverse magnetic (TM) hybrid modes, the waveguide design can offer high mode sensitivity (S_mode_) and evanescent field ratio (EFR). S_mode_ and EFR of 0.94 and 0.704, respectively, can be produced for the TE hybrid mode at optimum waveguide specifications, whereas S_mode_ and EFR of 0.86 and 0.67, respectively, can be obtained for the TM hybrid mode. At a 60% gas concentration, a 20 μm-long HPWG can dissipate power in the TE and TM hybrid modes by around 3 dB [84].

On photonic devices, waveguide structures limit light along predetermined routes and enable interaction between light and matter through an evanescent field [85]. For sensitive applications such as trace gas detection, waveguides still fall short of free-space optics. On-chip gas sensing is still in its infancy due, among other things, short optical pathlengths, weak interactions, and erroneous etalon fringes in spectrum transmission. Reference [86] describes a mid-infrared integrated waveguide sensor that satisfactorily overcomes these issues. In terms of the optical interaction per length, this sensor works with an evanescent field confinement factor of 107% in air, which not only complements but substantially beats free-space beams. The SEM image of the suspended rib waveguide is shown in Figure 4d [86]. With only a 2 cm long waveguide, the sensor’s performance at 2.566 μm demonstrated a 7 ppm detection limit for acetylene.

**Figure 5:**
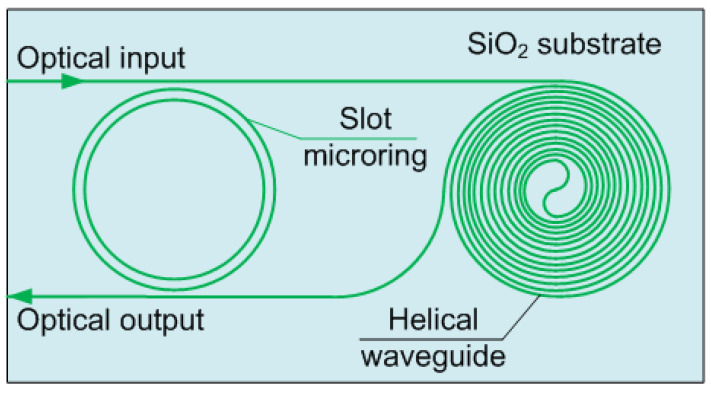
Schematic of slot microring gas sensor.

Toxic gas sensors based on integrated photonics are being actively researched. Sensors are described, in which the principle of operation is based on a change in the transmittance of the waveguide when the functional layer is exposed to the detected gas. SO_2_ and H_2_S detection sensors based on optical waveguides with an accuracy of 10 ppt are described in [87]. The device has a functional layer of 5,10,15,20-(tetra-4-aminophenyl) porphyrin (TAPP). When the film is exposed to SO_2_ and H_2_S gases, the Soret band shifts by 10 nm, and a new peak appears at 669 nm. The results showed that the limit of detection of the proposed sensor device was below 1 ppt. Environmental humidity did not significantly affect the TAPP-OWG device. The prepared TAPP-OWG sensor device showed high repeatability, high sensitivity, good reversibility, and fast response. These results indicate that the proposed sensor device is a promising option for detecting extremely low concentrations of SO_2_ and H_2_S gases in the atmosphere and the human body. However, the output intensity dynamic range reaches only 0.6 dB, which makes it difficult to use this device with photodiodes, so the use of resonator structures would be more appropriate. For example, the microring resonator described in [88] provides a sensitivity of 1.4 × 10^−2^ dB/ppm and a limit of detection of 700 ppb. However, the described scheme (Figure 5) is difficult to implement due to the presence of a helical waveguide.

Microring sensors are also frequently described for CO_2_ detection [89]. Silicon microring resonator with a radius of 10 um covered with a thin layer of the polyhexamethylene biguanide polymer, provided CO_2_ gas concentrations measuring in the 0–500 ppm range with a sensitivity of 6 × 10^−9^ RIU/ppm and a detection limit of 20 ppm. Table 2 presents the recent advances in evanescent field absorption-based optical waveguide gas sensors.

### 2.2. Water Quality Monitoring Sensors

Water is one of the most basic natural resources for human life and is essential to fundamental activities such as regulating the climate. When pollutants are added to water sources such as aquifers, lakes, rivers, seas, or groundwater, pollution ensues. Most of the garbage and contaminants thrown in water bodies each year are the result of human activity connected to industrial, touristic, and urban operations. Domestic garbage, waste from food processing, VOCs, heavy metals, pesticides, pollutants from livestock operations, and chemical waste are just a few examples of the various pollutants that end up in waterways. Both the chemical composition of the aquatic ecosystems and the loss of biodiversity has continuously deteriorated because of the outflow of these toxins and the heavy use of water resources. Another significant category of contaminants is pathogens, which are mostly linked to residential wastewater and cause illnesses that are transmitted through polluted water. Additionally, the dissemination of contaminants to people and the rest of the biota frequently occurs through the water. Examples include nutrient pollution, which promotes the growth of poisonous algae that harms other aquatic species, and pesticides that interfere with plant photosynthesis.

#### 2.2.1. Categories of Water Quality

Water may be categorized into surface water and ground water depending on where it comes from [94]. Both forms of water are subject to the danger of pollution by home, industrial, and agricultural processes that may use a variety of contaminants, including heavy metals, pesticides, fertilizers, toxic chemicals, and oils [95]. Four categories of water quality exist: drinkable (or potable), palatable, contaminated (or polluted), and infected [96]. The following are the most typical scientific definitions of various categories of water quality:

1. Potable water: it is suitable for drinking, tasting well, and being used in residential settings.
2. Palatable water: it is aesthetically appealing and considers the presence of substances that do not endanger human health.
3. Contaminated (polluted) water is water that is inappropriate for drinking or domestic use because it contains undesired physical, chemical, biological, or radioactive contaminants.
4. Infected water: this water contains harmful organisms. Water quality criteria can be classified as physical, chemical, or biological. Table 3 has an overview of them.

The preservation of water supplies and regulation of water quality are upcoming issues in Europe [97]. To prevent contamination by tiny organic molecules, particularly endocrine disrupting toxins, coastal areas, rivers, groundwater, wetlands, and especially drinking water necessitate monitoring. The proof-of-concept of immunochemistry for various applications has been shown via biosensors. It turns out that fluorescence detection-based optical approaches in particular can be successfully applied for the construction of quick, sensitive, affordable, and user-friendly analytical systems matching the standards set by European Community Directives and National Legislation [97]. Several analytical methods, such as mass spectrometry, capillary electrophoresis, liquid and gas chromatography, and spectrophotometry, were used to measure the dissolved oxygen, pH, turbidity, and organic/inorganic pollutants in water. Despite the great sensitivity and reproducibility of these methods, they are sometimes pricy, time-consuming, and need specialized equipment, experienced operators, and occasionally extensive pre-treatment processes that raise the possibility of sample loss and obstruct in situ real-time monitoring. Due to their benefits, including quick response times, low costs, portability, and the capacity to manage in situ in real time, fiber optic sensors have been rapidly developed for the detection of water quality [98,99,100,101].

Microcystins (MCs) in drinking water can be found using a surface plasmon resonance (SPR) biosensor that has been devised [102]. There have been evaluations of various assay formats. Microcystin-LR (MCLR) has been covalently immobilized on the surface of an SPR chip that has been functionalized with a self-assembled monolayer in the format that was chosen. This format is based on a competitive inhibition experiment. It has been determined how several aspects of sensor performance, such as antibody type and concentration, carrier buffer makeup, and blocking and regeneration solutions, affect sensor performance.

#### 2.2.2. Optical Sensors for Water Quality Monitoring

The fabrication and layout of a low-cost optical sensor for detecting the aquatic environment are given in [103]. The autonomous optical sensor is made to be reliable in harsh environments, portable, and easy to use. It is made up of two photodiode detectors that can measure the transmission and side-scattering of the light in the detector head and a multi-wavelength light source. As a result, the sensor is capable to provide qualitative information on variations in the optical transparency of the water. The results of laboratory testing to confirm color- and turbidity-related phenomena are discussed. The autonomous sensor was tested in the field in an estuary setting, and the findings are shown here. They demonstrate the sensor’s ability to recognize variations in opacity and color associated with possible pollution occurrences. This inexpensive optical sensor is used for environmental pollution warning to assist water monitoring programs, where a network of similar sensors might be established [103,104].

Kao and Hockham suggested the use of a guiding light in glass fibers in 1966 [105]. These fibers significantly improved our capacity to design optical fields with low loss, high compactness, flexible redirection, and a significantly longer light-matter interaction length, opening a new area for optical sensing known as optical fiber sensing. Although optical sensing technology, one of the most prominent sensor systems to date, has been around for 50 years and has been well-established, optical fiber sensing technology is actively evolving and continuously increasing to new application areas [106,107]. It has seen extensive application throughout the years in the industry, as well as in the surveillance of the environment, biology, chemistry, and health [108,109]. Because of recent significant advancements in optical fiber sensors for ocean detection, such as in situ measurement, invulnerability to electromagnetic interference, multiplex or distribution with high spatial resolution, lightweight, low cost, and low waterproof stipulation, the field has seen explosive growth [110]. Although not currently the standard technique for ocean detection, optical fiber sensors do provide several distinct benefits. For instance, when considering the temperature–depth profile taken by an autonomous underwater vehicle, an optical fiber grating temperature sensor’s measurement of the same sea area’s profile has a substantially greater spatial resolution [111].

In [112], it was suggested to use a straightforward and reliable hollow square-core fiber (HSCF) sensor to track the evaporation of ethanol–water mixes. With a maximum sensitivity of 387 nm/RIU, this sensor was able to track changes in the solution’s refractive index, enabling real-time monitoring of variations in the ethanol level during the evaporation process. For reasons of comparison, image analysis was also performed. The sensor’s capacity to track changes in sample temperature was also investigated. This measurement tool can be used to determine temperature compensation as well as infer some evaporation process characteristics.

The ocean has long been the subject of investigation and exploration because it plays a significant supporting role in the human habitat and influences variations in the Earth’s ecology and temperature. Several parametric analyses can be used to anticipate how the ocean will behave. Physical, chemical, topographical, and biological elements are typically included in ocean parameters [113]. For scientific researchers, precise readings of factors such as temperature, salinity, and pressure (TSP) will provide important information. TSP data, for instance, can be applied to calculate ocean acoustic transmission and other marine sensors as well as biogeochemistry and ecosystem background physical parameters [114]. Data from infrared or microwave satellites can be used to determine temperature and salinity (T&S) across a vast area [115]. Nevertheless, infrared light and microwaves are only suitable for acquiring the sea surface T&S due to their shallower penetration depths. Additionally, in situ measured data must be used to help the restoration of remote sensing data [116]. The advancement of maritime scientific study has been hampered by the lack of real-time observation data.

To address this issue, atmospheric and oceanographic researchers from the US, Japan, and other nations suggested the ARGO (Array for Real-Time Geostrophic Oceanography) project in 1998, which marked the beginning of a new era in the study of marine environmental observation [117]. After nearly 20 years of collaborative efforts by nations around the world, a global ARGO ocean observation network made up of 3000 ARGO surface buoys has been constructed. This network is capable of consistently obtaining the monitoring data of temperature, salinity, and depth to a depth of 2000 m in the deep ocean and providing free data to scientists around the world for research and application. The buoy cannot offer TSP data wherever due to its limited coverage of the ocean.

Fiber Bragg grating (FBG) sensors have been utilized extensively in recent years to detect a variety of ocean characteristics, and their architectures and encapsulation techniques are becoming more and more varied. In general, bare FBG is capable of sensing seawater temperature and pressure (T&P) since it is sensitive to ambient T&P. In 2014, Zhang et al. suggested a quick response ocean temperature sensor based on FBG with a reaction time of 48.6 ms and temperature sensitivity of 27.6 pm/°C for temperature sensing and measuring [118]. A graphene diaphragm-integrated silica FBG sensor head with a central wavelength of 1550 nm and temperature sensitivity of 13.31 pm/C was created by Ameen et al. in 2016 to measure temperature [119]. A polyimide (PI) tube-based Fabry–Perot interferometer (FPI) and FBG-based seawater temperature and pressure sensor are exhibited [120]. The sensitivity of the sensor is significantly increased because of the polymer’s better thermo-optical coefficient and greater flexibility compared to fused silica fiber. The temperature cross effect is compensated for using the FBG. The sensor’s observed sensitivities for temperature and pressure are 18.910 nm/°C and 35.605 nm/MPa, respectively [120]. A clad-less optical fiber sensor is proposed to analyze distilled water solutions polluted with methylene blue [121]. The manufactured sensor is based on evanescent wave absorption by the extracellular environment at the core–liquid cladding contact. The response of the optical fiber sensor is largely attributed to the methylene blue concentration including its refractive index. The study revealed that the developed sensor offers a considerable response to the concentration range of 6–50 mg/L [121].

**Figure 6:**
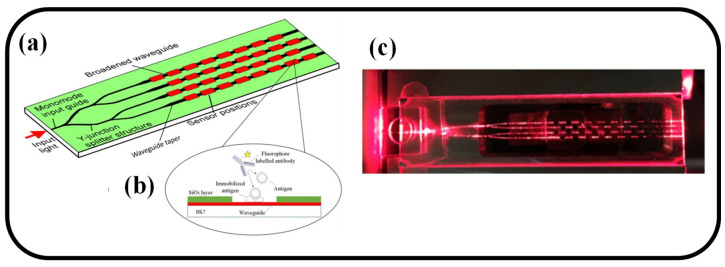
Integrated chip (**a**) an illustration of the sensor arrangement [122], (**b**) an illustration of one of the sensing patches in cross-section, highlighting the isolation layer, waveguide, and placement of the surface chemistry [122], (**c**) a visual representation of light movement along the waveguide chip [122].

An embedded channel waveguide-based fluorescent immunosensor with the ability to identify a maximum of 32 pollutants swiftly and concurrently is presented [122]. In a solid surface bioassay, waveguide tapers are used to increase the efficacy of excitation and collection of fluorescent signals in the presence of fluorophore photobleaching. This is the first instance of using that kind of waveguide immunosensor to identify microcystin-LR (MC-LR) in lake water under the optimized waveguide geometry. BSA-MC-LR conjugate was immobilized on the waveguide chip by (3-Mercaptopropyl) trimethoxysilane/*N*-(4-maleimidobutyryloxy) succinimide (MTS/GMBS), which was then shown to have homogeneous monolayer diffusion by atomic force microscopy. MC-LR in the sub-microgram range is present in all legitimate lake samples. The immunosensor’s application potential in the detection of MC-LR in actual water samples was confirmed by recovery rates ranging from 84% to 108% for the per liter range (for example, 0.5g/L) [122]. Following the arrangement illustrated in Figure 6a, a fiber-pigtailed waveguide chip is described that has a channel waveguide circuit that distributes excitation light to 32 different sensing patches on the surface. Figure 6b shows a cross-sectional view of one of the sensing patches together with the waveguide, isolation layer, and position of the surface chemistry; Figure 6c is an image of light traveling through the waveguide chip.

### 2.3. Indoor Environment Monitoring

The indoor environment quality (IEQ) refers to the circumstances within a structure and how they impact the occupants. Air quality, lighting, temperature, and ergonomics are some of these factors. IEQ improvements raise the building’s commercial value and increase the quality of life, health, and productivity of its residents. The major objectives of IEQ are to reduce the risk of health issues while at work and to create surroundings that are pleasant and conducive to productivity for individuals who work there. Since the worldwide pandemic’s consequences are still being felt, it is obvious why this program is now much more crucial than it was before.

#### 2.3.1. Volatile Organic Compounds Monitoring

**Figure 7:**
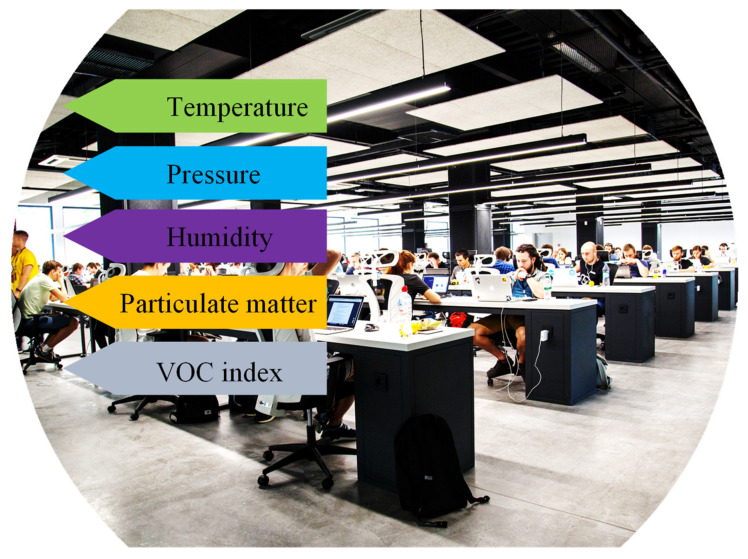
Indoor environment monitoring.

Volatile organic compounds (VOCs) are always present in nature and are crucial for plant-to-plant and plant-to-animals interactions. While most VOCs derived from natural and biological sources do not provide health or environmental risks, this is not the case for VOCs derived from human sources. VOCs produced by humans are typically linked to pollution, respiratory illnesses, and harm to vital organs and systems such as the liver, kidneys, and central nervous system. VOCs do represent a health concern, with some of them being very poisonous or causing long-term harm. Tracking the concentrations of VOCs in indoor and outdoor air as well as water systems is becoming more important in the present climate of escalating environmental and health concerns. Conventional techniques for detecting VOCs rely on mass spectrometry [123], gas chromatography [124], and high-performance liquid chromatography methods [125]. The practical distribution and implementation of these approaches for real-time water or air quality surveillance are hindered by their expense, time commitment, and bulkiness irrespective of the fact that they are precise and selective. Utilization of MEMS sensors [126] and MOS transistor-based sensors [127], which are based on the electrical resistance or resonant frequency shift when VOCs adsorb on the surface of a metal oxide or piezoelectric film, respectively, are other relatively common VOC detection methods. These sensors solve issues such as test expense, bulky equipment, and lengthy processing times in the laboratory of traditional approaches. However, their high operating temperatures—between 200 and 400 °C—require the use of a heater for on-chip temperature regulation, negating the benefits of both MOS and MEMS technologies’ low-power operation. Additionally, selectivity to certain compounds is constrained [128,129,130]. The indoor environment monitoring factors are shown in Figure 7.

A proposal is made for a VOC sensor architecture that addresses typical issues with photonic integrated sensors, such as reusability and specificity [131]. The suggested sensor includes chemically selective polydimethylsiloxane (PDMS) polymer cladding that encloses the waveguides and offers an extensible and permeable low-refractive index material. It is based on arrayed waveguide interference and is constructed on an SOI platform. In the context of environmental and public health protection, it is crucial to monitor the occurrence of certain volatile organic chemicals, which this cladding material serves as the chemical transducer element by altering its optical characteristics when in contact. The sensor works at room temperature, and several experiments using water, toluene, chlorobenzene, and hexane validated its selectivity. These tests also established the sensor’s durability. At a central wavelength of 1566.7 nm, verification with chlorobenzene resulted in a maximum spectral shift of around 22.8 nm. Additionally, at mass percent concentrations of chlorobenzene, a sensitivity of 234.8 pm/% was found, with a limit of detection of 0.24% m/m. The sensor’s thermal sensitivity was determined to be 0.9 nm/°C [131].

To investigate VOCs, silicon nitride (SiN) waveguide-based mid-IR sensors were developed and tested [132]. With a lower refractive index than common materials such as Si, SiN thin films made using low-pressure chemical vapor deposition (LPCVD) have a wider mid-IR transparent zone, which results in a stronger evanescent wave and increased sensitivity. Additionally, experimental proof of in situ monitoring of three VOCs (acetone, ethanol, and isoprene) was provided by measurements of their distinctive absorption at wavelengths between 3.0 and 3.6 µm. Due to its greater evanescent field than the Si waveguide, the SiN waveguide demonstrated a five-fold gain in sensitivity. As a result, the devised waveguide sensor has the prospective to be employed as a small device module that can monitor a variety of gaseous analytes for purposes in the areas of agriculture, environmental monitoring, and health monitoring.

**Figure 8:**
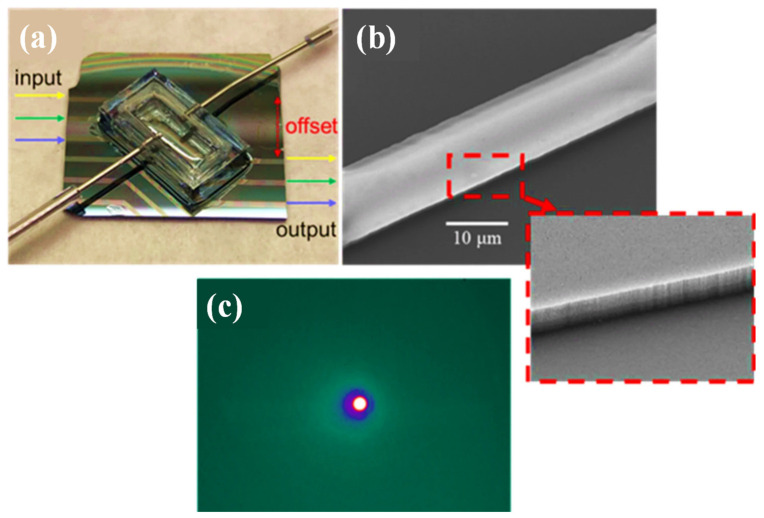
(**a**) Sensor device with PDMS chamber and SiN waveguides. Waveguides for the input and output had a 5 mm offset [132], (**b**) SEM image of the waveguide and inset shows the side wall [132], (**c**) near-field pattern of the fundamental mode [132].

The manufactured SiN waveguide sensor and associated PDMS gas chamber are shown in Figure 8a. To reduce influences from background light that was not connected to the waveguide, the input and output waveguides were offset from one another by 5 mm. An SEM view of the SiN waveguide and its die wall is shown in Figure 8b which indicates that the waveguide’s top surface and sidewall are both smooth. Figure 8c depicts a liquid-nitrogen-cooled mid-IR camera’s capture of the SiN waveguide’s waveguide mode at =3.3 µm. There was just one primary waveguide mode that was visible, and there was no dispersion over the whole field of vision. This shows that the input light was tightly contained within the SiN waveguide and that the offset design effectively reduced the background light in the substrate layer.

VOC detection is a subject of considerable interest with applications in many industries, including the food and chemical industries as well as environmental usage [133]. Over twenty years ago, optical fiber VOC sensors with novel and intriguing features that addressed some of the drawbacks of conventional gas sensors were introduced [134]. These sensors are a promising substitute for electronic ones in electrically noisy environments where electronic sensors cannot function properly because of their minimally invasive nature and the benefits that optical fiber provides, such as lightweight, passive nature, low attenuation, and the potential for multiplexing, among others [135]. There are certain evanescent wave sensor designs that do not employ a chemical dye [136]. For instance, it is possible to manufacture the cladding so that it is sensitive to particular organic vapors; another option is to taper the fiber, which results in a more delicate but sensitive sensor [137,138,139], or even use an optical fiber without a cladding so that the organic vapors themselves act as a cladding [140].

**Figure 9:**
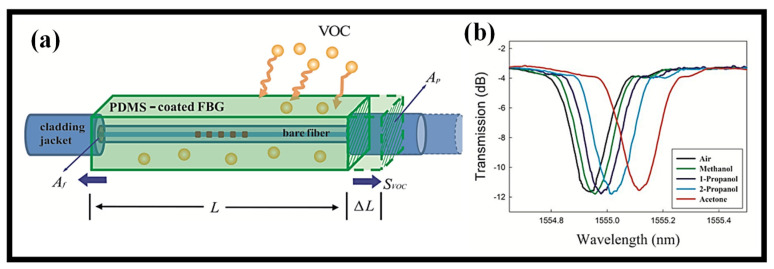
(**a**) PDMS-coated FBG sensor schematic diagram [135], (**b**) sensor spectral response for each VOC [135].

Most grating sensors that have been created and are now on the market are set up to detect temperature and strain. Meanwhile, Topliss et al. developed a unique VOC sensor design utilizing LPG in 2010 that was based on the wavelength interrogation approach [141]. The LPG fiber has been covered with a coating of calixarene, which demonstrates excellent sensitivity to aromatic chemicals such as benzene and toluene while being somewhat susceptible to other aliphatic hydrocarbons such as hexane. The monitoring of different VOCs using a unique selective chemical sensing method is described [135]. This method involves depositing PDMS on FBG structures. The sensor was built using a wavelength interrogation approach that took the use of the coating of PDMS on FBG’s swelling response when VOCs are present. The FBG receives a tensile tension from the swelling action. The swelling ratio of the sensing material has a significant impact on how much of this tensile tension is applied to the fiber. Therefore, by examining the Bragg wavelength shift of the PDMS-deposited FBG, the type and concentration of exposed VOCs may be precisely and selectively detected. Figure 9a displays the reported sensor’s schematic representation [135]. The sensor was evaluated using acetone, methanol, 1- and 2-propanol. A redshift in the resonance wavelength associated with each VOC is seen in Figure 9b [135]. The use of LPG coated with the novel sensing substance silk fibroin to create a VOC sensor for methanol detection is disclosed [142]. A thin layer of silk fibroin sensing film that was created on LPG using the drop-casting process makes up the sensor. Based on a wavelength interrogation approach, the sensor was used. As part of the setup, a sensitive manometer was installed in the gas chamber to monitor pressure changes brought on by the evaporation of the methanol that was supplied liquidly through the input. For methanol fluctuating between 80 and 100 mbar, the disclosed sensor shows a high sensitivity of 0.22 nm/mbar. This was the first time the function of the fibroin protein in the detection of VOCs with a good reversible response had been investigated [142]. Table 4 presents a few selective optical fiber grating-based VOC sensors recently proposed.

#### 2.3.2. Indoor Gas Monitoring

Optical fiber sensors may be applied in a variety of different situations. For these sensors to be marketed, they must adhere to a set of standards. The capacity of the sensor to distinguish one gas from others, also known as selectivity, is a crucial criterion when considering more detailed criteria for gas sensing. Extrinsic optical fiber sensors’ selectivity is primarily controlled by the light source wavelength; for intrinsic sensors, the selectivity is determined by the sensing material along with the chemical and physical characteristics of the matrix. Response time is yet another crucial factor. In terms of thickness and physical structure, it principally depends on the detecting chemical dye. The sensitive film needs to be diffused with the organic vapors that need to be detected. This makes it simple to obtain a thin, even layer of film. The recuperation time is another crucial factor. Response and recovery times should be as quick as feasible in online monitoring applications, although they are less important in other common applications, such as determining the maturation of fruit [143].

Applications where it is important to monitor the concentration of a specific gas or organic vapor typically call accurately and instantly for the deployment of high-selectivity sensors. This is crucial in regions containing poisonous gases such as NO_2_ [144], chloroform vapors [145], or dichloromethane [146], as well as highly explosive gases such as methane [56] or hydrogen [147]. In these situations, specialized modulation techniques are applied to improve the sensors’ selectivity and so counteract the effects of other gases. These applications commonly call for the monitoring of a large region; hence, these sensors are typically integrated into a network, making use of the multiplexation potential that optical fiber provides [148]. Evanescent wave sensors and extrinsic sensors based on wave spectral modulation are typically utilized when creating this type of network, executing a multi-point sensing network.

The Fiber Bragg Grating (FBG)-based sensors are the most viable technology for detecting minute changes in the environment among the wide range of optical fiber sensors. The process of creating FBGs involves introducing the core’s regularly varying refractive index throughout the length of optical fiber, which resonates at a wavelength commonly referred to as the Bragg wavelength. To put it simply, the FBG functions as an optical filter that reflects just a certain wavelength of light while transmitting all other wavelengths [149,150,151]. The advent of several optical fiber-based sensors has made great use of FBG ever since its creation in the late 1970s. They may be divided into two main types according to their grating period: short-period grating and long-period grating. In earlier reported articles, there is more specific information about how these fibers are made and how they work [152,153].

**Table 4:** Few selective grating-based optical fiber VOC sensors.

Grating Type	Sensing Material	Target VOC	Sensitivity	Operating Range	Reference
LPG	Zeolite imidazolate framework	Ethanol, acetone, and methanol	0.015 ± 0.001 nm/RIU for acetone and 0.018 ± 0.0015 nm/ppm for ethanol	987–19,700 ppm for acetone 1240 to 24,800 ppm for ethanol	[154]
LPG	PDMS	Xylene and cyclohexane	19 nm/50% for xylene	-	[155]
FBG	Diphenilalanine nanotubes	Methanol	(−7:3 ± 0:8) pm/(%*v*/*v*)	-	[156]
LPG	ZnO nanorod	Ethanol	Measure in refractive index variation	100 min exposure time	[157]
FBG	PMMA	Ethanol	Linear response for 3% concentration	-	[158]
D-shape FBG	PDMS	Dichloromethane, Acetone	4000 ppm, 6000 ppm	0–90,000 ppm	[159]
TFBG	Molecularly imprinted polymer	Ethanol, acetone, toluene	0.44 pm/ppm, 0.38 pm/ppm, 0.28 pm/ppm	0–17 ppm	[160]
LPG	PDMS	Acetone	9.4 × 10^−4^ ppm^−1^	-	[161]
4 FBG	Hydrophobic siloxane co-polymer	Hydrocarbons	-	-	[162]

### 2.4. Natural Disaster Monitoring

Natural disasters are catastrophic events that are caused by the planet’s natural processes. Just a few occurrences are earthquakes, tsunamis, floods, and storms. Earth has seen countless natural disasters in its ~4.5-billion-year existence. Some of these catastrophes have led to numerous mass extinctions and significant repercussions for numerous surviving species [163]. On the other hand, certain natural threats may be caused by or affected by anthropogenic factors [164]. Landslides can be brought on by a variety of activities, such as mining, agriculture, and deforestation [165]. Natural catastrophes can cause extensive harm. Animal habitats are destroyed by natural catastrophes such as wildfires, which can cause property damage and fatalities.

#### 2.4.1. Flood Monitoring

Monitoring the water level in flood-prone locations might provide authorities early notice of the kind of floods that recently destroyed many ASEAN (Association of Southeast Asian Nations) member states. Numerous sectors place a high priority on the monitoring of liquid levels, and the unique needs to monitor multiple containers of volatile fluids necessitate the use of secure and affordable sensor systems. Monitoring of the water level in catchment regions, monitoring of illegal rubbish and pollution in canals (which may be deduced by an unexpected rise in water level), and early drought warning are additional applications where liquid level monitoring is needed (when water levels drop to a minimum level). Optical fibers are increasingly being used in sensing-related applications [166,167]. These well-known benefits comprise their immunity to electromagnetic interference, multiplexing capabilities, distributed sensing, non-intrusiveness, lightweight design, and lack of explosion danger due to their natural spark-free nature. The optical fiber-based level sensing system can be categorized in several ways. The sensors may be divided into three categories: continuous [168,169] and discrete/point measurements [170,171,172], intrusive [170,171,172], and non-intrusive types [168,169], and all-fiber types (which do not need attachment) [173,174].

Glass-based optical fibers were utilized in [174,175], and extra caution must be exercised while bending the fiber to construct a “U”-shaped sensor tip to avoid fiber breakage. Additionally, the bending radius (for a given fiber diameter) may be constrained, making it difficult to get the best sensitivity [176]. A method to bend glass fiber to the right radius has been described in another study as heating the fiber [177]. In a subsequent study employing glass optical fibers, the sensing probe was built by using a fusion-splicer to join three fibers [172]. The intricacy of the sensor’s construction is increased by the need for precise current splicing selection throughout this manufacturing process to get the correct sensor head profile and performance. However, in the current work, the process for creating the detecting tip is much more streamlined. The plastic optical fibers (POFs) may be easily twisted into the desired shape without running the risk of breaking, eliminating the need for fusion-splicing or heating during the sensor’s fabrication.

**Figure 10:**
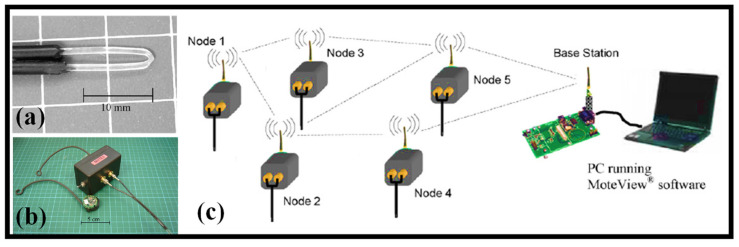
Flood monitoring (**a**) image of the modified U-shaped sensor profile [178], (**b**) photo of the wireless POF prototype and the MICA2DOT unit [178], (**c**) representation of the universal wireless POF sensor system [178].

It is suggested to create a wireless mesh network using POF sensors for a remote flood monitoring system [178]. The wireless mote, which is made up of a system of MICA2DOTTM units, served as a platform for tracking and recording the signal from the POF sensors and wirelessly transmitting that data to a base station. The integrated wireless POF sensor unit prototype has been built, making it possible to remotely deploy the autonomous unit at as many monitoring sites as necessary. The V-shaped probe is shown in Figure 10a. Four of these wireless optical fiber mote sensors were employed in a 24 m by 10 m by 0.9 m wave basin during a flood monitoring simulation to find the growing water level there. The photograph of the unit is shown in Figure 10b. The optical signal’s loss of total internal reflection when the sensor probe makes contact with the liquid is the basis for the POF sensor’s well-known sensing mechanism. The probe profile employed in this work varies from optical fiber-based sensors previously described in the literature in terms of its simplicity in design while demonstrating an excellent signal intensity loss ratio even without the requirement for further modifications to the probe such as optical prisms [178]. The schematic of the generic wireless POF sensor system is shown in Figure 10c.

#### 2.4.2. Earthquake Monitoring

To research undersea earthquakes and the Earth’s interior, seafloor geophysical apparatus is difficult to deploy and maintain. To close the data gap, new fiber-optic sensor technologies that can use underwater communications cables are being developed. By observing the polarization of ordinary optical telecommunication channels, seismic and water waves along a 10,000 km undersea cable between Los Angeles, California, and Valparaiso, Chile, are detected [179]. Along the wire, many moderate-to-large earthquakes have been picked up in the 10-millihertz to 5-hertz frequency. Ocean swell pressure signals are detected in the main microseism band, suggesting the possibility of tsunami sensing. This technique is very scalable for transforming worldwide underwater cables into continuous real-time earthquake and tsunami observatories since it does not call for specific equipment, laser sources, or dedicated fibers [179].

In general, sensor systems for monitoring seismic activity can be divided into two main classes: acoustic (registration of infrasound and ultrasound) [180] and radiation (based on determining the concentration of radon gas) [181]. The most developed method [180] is DAS (“distributed acoustic sensing”) technology. DAS uses fiber optic cables installed specifically for research purposes [182] or existing “dark” fiber of the optic networks (unused fibers that were originally installed for communication) [183]. In addition, to increase the sensitivity, optical fibers are supplemented with fiber Bragg gratings (FBG) [184]. Optical beams passing through an optical fiber are reflected at locations where the properties or geometry of the optical fiber changes, which occurs when it is subjected to local vibrations in the environment.

**Figure 11:**
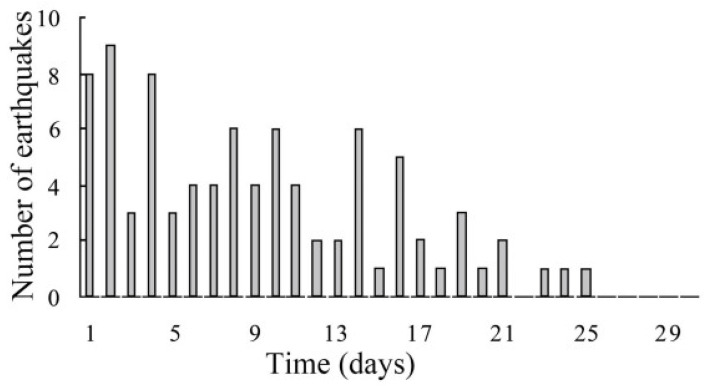
Time distribution of the anomalous infrasound signals before the earthquakes during 2002–2009 [185].

Earthquake prediction is a difficult task. One of the signs of an approaching earthquake is infrasonic waves that occur when cracks appear in rocks. The growing amplitude of acoustic waves, coinciding with the expansion of cracks and an increase in the zone of their propagation, is detected long before the main earthquake. It was shown that in almost 76% of large earthquakes (magnitude M more than 7) around the world in the period from 2002 to 2009, anomalous infrasound was recorded for 1–9 days immediately before the earthquake [185]. In some cases, it was discovered more than two weeks before the main seismic shocks (Figure 11).

Infrasound is the basis for the development of early warning signals for several dangerous processes. For example, landslides are among the processes characteristic of rugged terrain and can pose a serious threat to settlements and infrastructure. It has been shown [186] that detection methods based on infrasound monitoring can generate a mudflow warning signal 10–30 s before it starts. In many cases, such a warning can provide even up to five minutes, during which evacuation measures can be taken. It should be noted that an obligatory factor in infrasound landslide monitoring is signal processing and elimination of the extraneous infrasound influence, for example, from such sources as lightning or vehicles. The described method has been improved in [187], where the system of early detection of landslides based on the principles of determining their different phases was demonstrated. It made it possible to detect the first signs of approaching landslides 100–150 min before their start.

**Figure 12:**
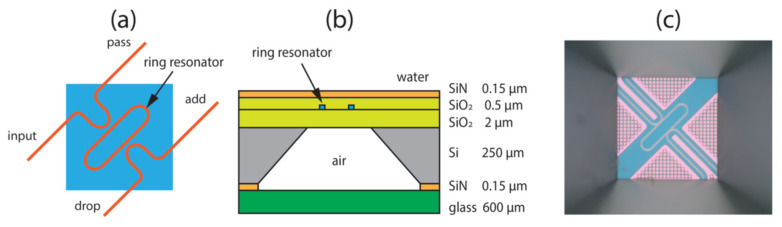
(**a**) Schematic of the RR ultrasound sensor: a RR on a square membrane (blue). (**b**) Cross-section of the membrane region, showing the various layers and their thickness. Membrane thickness is 2:65 μm. A glass platelet seals the air cavity under the membrane. (**c**) Microscope image of the membrane with the RR, taken from below. The membrane size is 84 μm × 84 μm [190].

**Figure 13:**
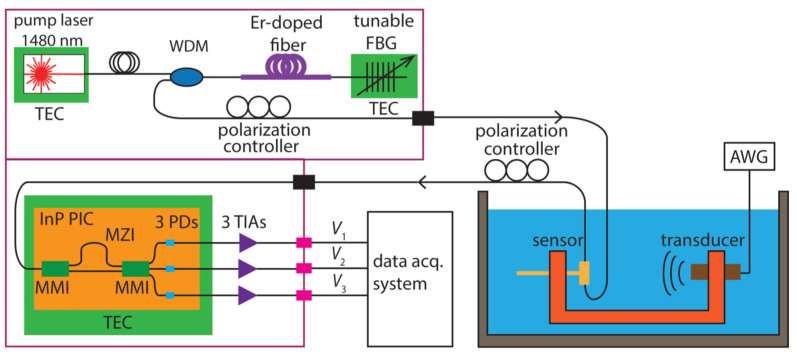
MediGator (**left**) and experimental setup (**right**). The MediGator comprises the tunable light source of high brightness (**upper** part) and the photonic integrated circuit of MZI and PDs (**lower** part). Each PD is connected to a TIA. (TEC, thermoelectric cooling; WDM, wavelength division multiplexer; FBG, fiber Bragg grating; PIC, photonic integrated circuit; PD, photodetector; TIA, trans-impedance amplifier; AWG, arbitrary waveform generator; MMI, multi-mode interferometer) [190].

High-frequency sounds are generated by several processes in rocks. Ultrasound in nature is less studied than audible frequencies but is considered to be the result of the gradual destruction of rocks, for example, as a result of freezing processes. The short-term release of acoustic energy associated with crack growth generates high frequencies [180]. Weber et al. present an additional case study involving field monitoring of ultrasonic vibrations [188]. Abnormal levels of ultrasound were detected 8 days before the main shocks. In this work, measurements were made using a piezoelectric element. However, in [189], a system was demonstrated using phase-shifting FBGs. In [190], a compact photonic integrated interrogator for a ring-resonator (RR) ultrasonic sensor is presented (Figure 12). This system (Figure 13) consists of a special light source and a Mach–Zehnder (MZI) InP (indium phosphide) interferometer with a 3 × 3 multimode interferometer or multimode interference coupler (MMI) [190].

An alternative method for predicting seismic activity is to determine the concentration of radon gas. It is known that a high concentration of radon indicates a growing earthquake [181]. Optical fiber is used as a tool for detecting alpha particle emission and hence can be used to determine radon concentration. Gas detection is achieved by measuring the damage and degradation of the fiber optical transparency as a function of the time of exposure to alpha rays emitted by radon gas. In [191], the concentration of radon was determined using a fiber-optic detector consisting of an FBG array and Fabry–Perot resonators, which made it possible to increase the sensitivity of the system. This method allows you to predict an earthquake in 1–2 weeks before the main shocks.

#### 2.4.3. Volcanic Eruptions

Recent years have seen a tremendous improvement in our understanding of physical processes leading up to and during volcanic eruptions. Volcanologists are unable to deduce nuanced triggering mechanisms of volcanic occurrences, nevertheless, due to ambiguities regarding underlying structures that distort observable signals and undetectable activities inside the volcano. It is shown that using optical fibers and distributed acoustic sensing (DAS) enables distant identification of volcanic occurrences and imaging of obscure near-surface volcanic structural features [192]. Using a 2D template matching between selected and predicted wave arrival periods, strain signals connected to explosions are identified and their source is determined. It was discovered that a scoria layer with spatially varying thickness contained evidence of non-linear grain interactions. The wavefield separation makes it possible to gradually examine the ground’s reaction to different excitation techniques. It was discovered that very minor volcanic eruptions connected to fluid movement and degassing. These findings serve as the foundation for better DAS-based volcanic surveillance and hazard identification [192].

#### 2.4.4. Storms Monitoring

Devastating storms and strong seasonal winds can harm high-voltage transmission towers in coastal locations. Systems for real-time condition monitoring allow for the early detection and intervention of severe and critical damage. An all-optical fiber sensing system is being researched for the potential to monitor transmission tower vibration caused by wind in real time [193]. The thin-core fiber with tilt fiber gratings carved into its core is coaxially spliced to a lead-in single-mode fiber to create the optical sensing probe. Well-packaged and immediately placed on the transmission tower is the sensing probe. One wavelength-separated spectral signature from the cladding modes and one from the core-guided mode are concurrently provided by the sensor. The optical power of the cladding modes displays the transmission tower’s real-time vibration information, including its amplitude and frequency. Core mode’s capability offers in-situ temperature measurement as well as intrinsic self-calibration to eliminate light source intensity and optical transmission loss changes. During field tests, the viability of the all-optical fiber sensing system is shown. It was shown that there is a consistent and repeatable association between the transmission tower’s vibrational acceleration and the wind speed on a real-time basis [193].

#### 2.4.5. Landslide Monitoring

**Figure 14:**
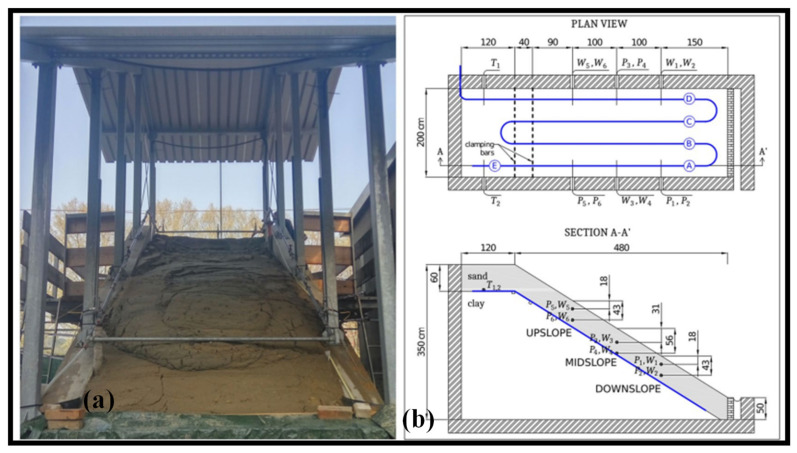
Landslide monitoring, (**a**) the large-scale physical replica underwent the and slide during testing [187], (**b**) views from the top and sides of the instrumented fume show the clamping bars as vertical dashed lines in the top image [187].

An optical fiber buried in a large-scale physical model of a slope is subjected to landslide-induced strain measurements using a distributed optical fiber sensor system. Using optical frequency domain reflectometry, the fiber sensor cable is placed at the predetermined failure surface and inspected [187]. Up to the advent of the slope collapse, the strain evolution is measured with centimeter spatial precision. For comparison and verification, standard legacy sensors measuring pore water pressure and soil moisture are put at various depths and locations along the slope. With previously unheard-of clarity and knowledge, the development of the strain field is connected to the dynamics of landslides. In reality, the experiment’s findings clearly define multiple stages in the evolution of the landslide and demonstrate that optical fibers may recognize early warning indicators of failure long before a collapse occurs. This opens the door for the creation of more potent early warning systems. The experiment was conducted inside a 6-by-2 m reinforced-concrete structure that served as a large-scale physical replica of a slope as shown in Figure 14a. The upper plot of Figure 14b depicts how the sensing cable is set up. The cable, which is roughly 35 m long, is set up in four main straight spans that go longitudinally down the slope and are spaced, respectively, 0.25, 0.75, 1.25, and 1.75 m from the left lateral wall. At the top of the slope, two aluminum profiles connected to the lateral walls are mechanically fastened onto the straight spans, denoted in the illustration by the capital letters A, B, C, and D. The higher profile is at the top of the slope, while the lower profile is 0.4m down the slope. These are shown in the picture as vertical dashed lines that lie at the interface of the strata. At the toe, the cable is unrestricted. Measured and analyzed only were the stresses exerted on the fiber over the four straight spans between the top bar and the toe. To adjust the results for the effects of temperature fluctuations, additional measurements were made along span E (the continuation of span A) [187].

## 3. Outlook and Conclusions

Concerns about global environmental challenges, such as the alarming increase in pollution of our oceans, waterways, land, and air, are becoming more and more prevalent in contemporary society. Environmental pollution has evolved into more than a health concern because of global industrialization and mass consumption patterns; it now represents a danger to whole ecosystems. It is critical to comprehend its causes and mitigation strategies. Adequate and timely environmental data are required for risk forecasting and early warning for environmental disasters including floods, severe weather, and volcanic eruptions [96,184]. It is necessary to gather environmental data to obtain this knowledge.

The usage of fiber-optic sensing techniques has expanded over the past 30 years to a wide range of applications in several scientific fields. With the introduction of practical, low-loss single-mode fiber created for the telecommunications sector, the technology and its related equipment commenced emerging as a separate field in the middle to late 1980s. This made it possible to transport information across vast distances for communications and environmental parameter sensing. The fiber-optic gyroscope may have been the first widely used application of fiber-optic sensors, and optical time-domain reflectometry, which allowed for long-range distributed sensing based on time-of-flight signals reflected from perturbations along the length of the fiber using a brief laser pulse, came in second. These disturbances may be mechanical or environmental such as temperature variations. The most frequently used fiber-optic sensing technique for environmental applications is fiber-optic distributed temperature sensing (DTS or FO-DTS) (especially in hydrologic applications) [194,195]. Although FO-DTS systems were commercialized in the 1980s, it appears that at the time of industrialization, environmental applications were not considered. Early literature anticipated the technology’s potential use for energy management, fire detection, process control engineering, and other significant industrial applications. It was initially deployed in the oil and gas sector in the late 1990s. Borehole monitoring with FO-DTS started to be employed in environmental applications not long after it became standard procedure in the oil and gas sector. Hurtig et al. used an FO-DTS system in combination with hot- and cold-water injections to identify fractured rock flow via a borehole [196].

Additionally, there is unexplored potential for fiber-optic chemical sensors in environmental applications. Although they are presently underutilized, they are also not unheard of for environmental applications and hold promise for assessing a wide range of water, soil, and air quality indicators. The miniaturized distributed oxygen sensor created by Brandt et al., which combines an off-the-shelf fiber-optic oxygen transmitter with a polymer optical fiber coated with oxygen-sensitive dye in a tubular sensing element, is one contemporary use of this technology. Oxygen profiles in the streambed were acquired, and the top of the anoxic zone was detected by placing the tubular sensing element into the hyporheic zone of a streambed and advancing the fiber vertically throughout the tube. Comparable sensors could be developed to assess various other parameters. For instance, Qian et al. [197] discuss different approaches to creating fiber-optic sensors for measuring salinity, and Palmieri and Schenato describe the use of Rayleigh backscatter measurement in doped fibers to sense temperature and strain along a fiber, with measurements on the order of centimeters possible [198].

For environmental protection and manufacturing safety, portable or even on-chip hazardous gas detection is important. However, because optical sensing systems are often built using separate optical components, they are inappropriate for situations where a high level of mobility is required [15]. Refractive index sensing and optical absorption sensing are two of the many methods based on optical waveguides that have been suggested to measure gas concentration. Refractive index sensing works by monitoring changes in the refractive index of the gas-sensitive material such as polymer, which would affect the output light’s frequency or phase. Since each gas has a distinct absorption spectrum, waveguide sensors based on optical absorption spectroscopy are more selective than the wavelength interrogation method. The concentration of the gas can be estimated by measuring the light attenuation that occurs when the light of a certain wavelength passes through the gas [14].

After carbon dioxide (CO_2_), methane (CH_4_) is recognized as the second-most significant greenhouse gas, causing roughly 20% of the global warming brought on by greenhouse gas emissions [83]. The use of fossil fuels including coal, oil, and natural gas has led to an increase in atmospheric CH_4_ concentration in recent years. Additionally, CH_4_ is explosive when combined with 5–15% of air, which might pose a risk to industrial safety. Therefore, effective CH_4_ concentration level monitoring is required for both ecological sustainability and product safety. On-chip optical waveguide sensors offer a smaller device footprint than sensing systems based on separate optical components and can combine the light source and detector on a single chip. When compared to the absorption in the near-infrared, CH_4_ exhibits a greater absorption in the mid-infrared between 3.3 μm and 7.6 μm [17]. Due to their low absorption loss, chalcogenide glasses are typically employed in the mid-infrared range. Due to its transparency in the CH_4_ absorption waveband (3.2–3.45 μm), SOI is another promising material platform for mid-infrared CH_4_ sensing in addition to chalcogenide glasses. An improved SOI waveguide’s cross-section architecture can result in a greater power confinement factor.

Photonic sensors are vital for environmental monitoring and are extensively researched. For specific needs, both photonic integrated circuits and optical fiber-based sensors can be employed for environmental monitoring. However, there are certain instances such as water quality monitoring and natural disaster situations where integrated waveguide sensors are not suitable and only optical fiber-based sensors can be employed. We believe that photonic sensors should be extensively researched as they play an important role in the early warning and preservation of the global environment.

## Figures and Tables

**Table 1:** Optical fiber-based gas sensors.

Material for Selective Sensing	Gas Detection	Sensitivity	LOD (%)	Range (%)	References
Crytophane E	Methane	−1.6 nm/%	0.06	0–5	[59]
Graphene + Ag	Methane	0.34 nm/%	0.1	0–3.5	[60]
Crytophane A	Methane	6.39 nm/%	0.015	0–3.5	[61]
Carbon nanotubes	Carbon dioxide	0.04 nm/%	0.05	0–100	[62]
Nickel oxide and reduced graphene oxide	Carbon dioxide	1400 a.u/%	0.0005	0–0.05	[63]
Carbon nanotubes and polyallylamine	Carbon dioxide	0.1 nm/%	0.01	0.1–0.4	[64]
Divinylbenzne and siloxane polymer	Nitrous oxides	5 × 10^7^ dB/%	10^7^	0–1.8 × 10^−6^	[65]
Carbon nanotubes, polyethyleneimine, Au	Nitrous oxides	0.05 nm/%	0.0109	0–100	[66]
	Nitrous oxides	82 μV/%	0.001	0–2.5	[67]
Graphene oxide	Water vapor	0.349 dB/%	0.2	30–77	[68]
Chitosan	Water vapor	0.107 nm/%	0.1	30–77	[69]
Graphene quantum dots	Water vapor	0.567 nm/%	0.05	11–85	[70]

**Table 2:** Evanescent field absorption-based optical waveguide gas sensors.

Platform	Sensing Mechanism	Waveguide Type	Target Gas	Wavelength (μm)	Reference
SOI	Evanescent field absorption	Strip, rib, slot	Carbon dioxide	4.23	[90]
SOI	Evanescent field absorption	Hybrid plasmonic	Methane	3.392	[16]
SOI	Evanescent field absorption	Suspended membrane	Methane	3.39	[91]
SOS	Evanescent field absorption	Rib	Carbon monoxide	4.67	[14]
SOI	Evanescent field absorption	Strip	Methane	3.39	[17]
SOI	Evanescent field absorption	Ridge	Methane	3.291	[92]
Chalcogenide or porous germanium	Evanescent field absorption	Ridge	Carbon dioxide and methane	4.7 and 7.7	[93]

**Table 3:** Types of water quality parameters.

Biological Parameters	Chemical Parameters	Physical Parameters
Bacteria	Acidity	Temperature
Algae	Alkalinity	Color
Viruses	Chloride	Odor
Protozoa	pH	Taste
	Hardness	Electrical conductivity
	Fluoride	Turbidity
	Copper and Zinc	
	Radioactive substances	
	Toxic (organic or inorganic) substances	
	Dissolved oxygen	

## Data Availability

Not applicable.
